# Distribution of extended-spectrum β-lactamase producing *Escherichia coli* genes in an integrated poultry-fish farming system in Bogor, Indonesia

**DOI:** 10.14202/vetworld.2024.1596-1602

**Published:** 2024-07-26

**Authors:** Kusuma Sri Handayani, Agus Setiyono, Denny Widaya Lukman, Herwin Pisestyani, Puji Rahayu

**Affiliations:** 1 Animal Biomedical Science Study Program, School of Veterinary Medicine and Biomedical Sciences, IPB University, Bogor, Indonesia; 2 Bogor Agricultural Development Polytechnic, Bogor, Indonesia; 3 Division of Pathology, School of Veterinary Medicine and Biomedical Sciences, IPB University, Bogor, Indonesia; 4 Division of Veterinary Public Health and Epidemiology, School of Veterinary Medicine and Biomedical Sciences, IPB University, Bogor, Indonesia; 5 Animal Products Quality Testing and Certification Center, Directorate General of Livestock and Animal Health, Ministry of Agriculture, Bogor, Indonesia

**Keywords:** *bla*CTX-M, *bla*OXA-48, *bla*SHV, *bla*TEM, integrated poultry-fish farming system

## Abstract

**Background and Aim:**

The excessive use of antimicrobials in livestock farming leads to the emergence and dissemination of antimicrobial-resistant organisms. This study aimed to detect extended-spectrum β-lactamase (ESBL)-producing *Escherichia coli* genes in integrated poultry-fish farms in Bogor, Indonesia.

**Materials and Methods:**

A total of 256 samples were collected from six poultry-fish farms. One hundred and seventy-five chicken cloaca swabs, 60 fish skin swabs, six pond water samples, and 15 farmer’s hand swabs. ESBL-producing *E. coli* was confirmed through double-disk diffusion. The specific primers and probe genes for quantitative polymerase chain reaction detection of ESBL-producing *E. coli* targeted *bla*TEM, *bla*CTX-M, *bla*SHV, and *bla*OXA-48 genes.

**Results:**

Among the 256 samples tested, 145 (56.6%) were positive for *E. coli*, and 67.6% (98/145) were identified as ESBL-producing *E. coli*. The most ESBL-producing *E. coli* isolates were obtained from chicken cloaca (78.3%, 72/92), followed by pond water (66.7%, 4/6), fish skin (47.6%, 20/42), and farmer’s hand swabs (40%, 2/5). About 100% of the isolates carried the genes *bla*TEM and *bla*CTX-M, whereas 17.3% and 24.5% carried *bla*SHV and *bla*OXA-48, respectively.

**Conclusion:**

ESBL-producing *E. coli* genes were investigated in chicken cloaca, fish, pond water, and farmers’ hands within an interconnected poultry-fish farming operation. The ESBL-producing *E. coli* in chickens can transfer resistant genes to aquatic environments. The transfer could harm other aquatic species and food chains, potentially threatening human health.

## Introduction

The issue of antibiotic resistance in bacteria poses significant threats to both human and animal health, including global food security [1]. Resistance to antibiotics can make certain harmful bacteria even more hazardous in the absence of prevention methods [2]. The generation of extended-spectrum β-lactamase (ESBL) by Enterobacteriaceae, including *Escherichia coli* and *Klebsiella pneumoniae*, intensifies this challenge [3]. *E. coli*, with its presence in human and animal gastrointestinal tracts, genetic flexibility, and adaptability to diverse environments, significantly contributes to the spread of antibiotic resistance dissemination [4]. *E. coli*, found in diverse habitats and can horizontally and vertically transfer antibiotic resistance genes through mobile genetic elements and cell division, serves as a reservoir for these resistance traits. Antibiotic resistance rapidly spreads in animals, the environment, and human communities because of this phenomenon [5]. Antimicrobials are widely used to improve livestock health and productivity, but they pose a risk of antibiotic resistance, particularly in systems in which multiple species are kept simultaneously [6]. Overuse of antimicrobials in animals and humans causes antibiotic resistance in pathogenic bacteria and endogenous microflora. Resistant bacteria can be transmitted to humans through various pathways, such as direct contact with animals, feces, and the food chain [7].

In an integrated poultry-fish farming system, poultry and fish are co-reared. In this system, poultry directly defecates into the pond. Manure is used as organic fertilizer to support the development of organisms and grow natural food, which is directly consumed by fish, including the feed that falls into the pond [6, 8], facilitating the transfer of antibiotic-resistant bacteria from chickens to the aquatic environment. The accumulation of antimicrobials, their residues, and antimicrobial-resistant microbes in livestock-rearing systems necessitates caution due to potential public health concerns. This promotes the growth of antibiotic-resistant bacteria [9].

Although several studies [10–12] have been conducted on ESBL-producing *E. coli* in Indonesia, there is insufficient data on the presence and spread of these bacteria in integrated fish-poultry farms. This study aimed to identify ESBL-producing *E. coli* genes in Bogor’s integrated poultry-fish farms in Indonesia to enhance antibiotic resistance control efforts.

## Materials and Methods

### Ethical approval

The Animal Ethics Commission of the Faculty of Veterinary Medicine and Biomedical Sciences (IPB University number 134/KEH/SKE/X/2023) approved the study protocol.

### Study period and location

The study was conducted from October 2023 to January 2024, with samples collected from six integrated poultry-fish farms in Bogor, Indonesia. The isolation and identification of ESBL-producing *E. coli* were conducted at the Veterinary Public Health Laboratory, Faculty of Veterinary Medicine and Biomedical Sciences, IPB University. Genotype identification of ESBL-producing *E. coli* was conducted at the Animal Product Quality Testing Laboratory and Certification Center, Ministry of Agriculture, Indonesia.

### Sample collection

Samples were collected from six farms with integrated poultry-fish systems at different locations in Bogor, Indonesia. A total of 256 samples were collected, including chicken cloaca swabs (n = 175), fish skin swabs (n = 60), pond water (n = 6), and farmer hand swabs (n = 15).

Swabs were collected from poultry in six coops at various locations, and fish skins from catfish, carp, and shark catfish are located in ponds beneath the coops. 1 L water samples were collected from each of the six ponds. Swabs were collected from farmers who had contact with poultry and fish in the integrated farms. Sampling was performed at a single location within the time frame of 06.00-09.00 am. The collection method followed the standard microbiological analysis protocol [13]. Each sample was transferred into a 10 mL buffered peptone water filled sterile test tube for transport. A separate sterile glass bottle was used to collect water samples at each pond location. A cool box (4°C) was used to transport all samples to the laboratory immediately after collection.

### Isolation and identification of *E. coli*

Samples were incubated in EC Broth (Oxoid, UK) at 45.50°C for 48 h after homogenization using a tube shaker. Samples showing both turbidity and gas in the Durham tube were positive. Subsequently, samples were inoculated into Petri dishes containing tryptone bile X-glucuronide (TBX) agar (Oxoid) and incubated at 36 ± 1°C for 18–24 h. A total of five bluish-green colonies suspected to be *E. coli* on each TBX agar medium were transferred using an inoculation loop and inoculated into Mac Conkey agar containing cefotaxime 4 μg/mL. This was followed by incubation at 36 ± 1°C for 18–24 h, with *E. coli* colonies exhibiting small, slimy, well-demarcated, and red color. An indole biochemical test was conducted through sulfide indole motility media incubation for 18–24 h at 37°C, and positive results were shown by the formation of a red ring color after adding Kovacs reagent [14, 15]. As a positive control, *E. coli* isolate ATCC 25922 was used, which was identified as pure *E. coli*.

### ESBL double-disk confirmation test

ESBL-producing *E. coli* was confirmed using the double-disk diffusion method on Mueller Hinton agar (MHA) (Merck 1.05458.0500, Germany), following the Clinical and Laboratory Standards Institute guidelines [16]. Pure cultures were prepared as a suspension equivalent to 0.5 McFarland turbidity (1–2 × 10^8^ colony forming unit/mL). The Petri dish was evenly coated with the cultured sample after being swabbed with a sterile cotton swab and streaked onto MHA. Paper disks containing antibiotics (cefotaxime 30 μg, cefotaxime clavulanic acid 30 μg, ceftazidime 30 μg, and ceftazidime-clavulanic acid 30 μg) were placed on MHA at a distance of 25–30 mm and incubated at 35°C for 24 h. Positive ESBL results were determined by observing the inhibition zone around the antibiotic disks. ESBL-producing strains were characterized when the inhibition zone for cefotaxime or ceftazidime disks combined with clavulanic acid, compared with the diameter of the disk zone without clavulanic acid, was ≥5 mm [17, 18].

### Genotype detection of ESBL-producing *E. coli*

The initial process of ESBL-producing *E. coli* gene detection was bacteria DNA extraction, which included isolate preparation, cell lysis, DNA binding, washing, and elution. Pure *E. coli* isolates were transferred using an inoculation loop from culture media into microtubes containing 1 mL of sterile phosphate-buffered saline (PBS) until a turbidity of 0.5 McFarland standards was reached. The suspension was centrifuged at 13,000× *g* for 5 min, and the supernatant was discarded, followed by 200 μL of sterile PBS was added to the bacterial pellet. After homogenization using a vortex mixer, the suspension was centrifuged at 13,000× *g* for 5 min. Subsequently, 200 μL of Fast Lysis Buffer was added and placed in a Thermo Mixer (Eppendorf, Germany) for heating at 100°C at 800 rpm for 10 min, followed by cooling for 2 min at room temperature (15–25°C). The resulting suspension was centrifuged at 13,000× *g* for 5 min, and the final extraction stage included transferring 100 μL of the supernatant containing DNA into a 2 mL microtube and incubating at 80°C until further analysis. Furthermore, quality control of the extracted DNA was performed by testing the concentration of DNA purity using a nanodrop spectrophotometer (Thermo Fisher Scientific, Massachusetts, USA), which ranged from 1.8 to 2.0 (A260/A280).

The quantitative polymerase chain reaction (qPCR) Taqman probe method was used to detect ESBL-producing *E. coli* genes. DNA amplification was performed for 40 cycles with denaturation for 10 s at 95°C, annealing for 60 s at 54°C, and extension for 90 s at 72°C. The genes *bla*TEM, *bla*CTX-M, *bla*SHV, and *bla*OXA-48 were used, whose primers and probes are listed in Table-1 [19, 20].

**Table-1:** Specific primers and probes for detecting ESBL-producing *Escherichia coli* genes.

Primers and probes	Sequence 5’- 3’	Product size (bp)	Reference
qTEM-F	CGGATGGCATGACAGTAAGA	101	[19]
qTEM-R	GTAAGTTGGCAGCAGTGTTATC		
qTEM-P	Hex-TGCAGTGCTGCCATAACCATGAGT-BHQ1		
qCTX-M-F	CTATGGCACCACCAAYGATA	86	[19]
qCTX-M-R	TTGAGGCTGGGTRAARTARG		
qCTX-M-P	TAMRA-ACCAGAAYCAGCGGCGCACGAY-BHQ2		
qSHV-F	TGGATGCCGGTGACGAA	90	[19]
qSHV-R	CAAGGTGTTTTTCGCTGACC		
qSHV-P	CTGGAGCGAAAGATCCACTATCGCCA-BHQ1		
qOXA-48-F	GTAGCAAAGGAATGGCAA	100	[20]
qOXA-48-R	CCTTGCTGCTTATTGTCA		
qOXA-48-P	FAM-TCC(+A) GA(+G) CA(+C) AA(+C) TACG-TAMRA		

ESBL=Extended-spectrum β-lactamase

### Statistical analysis

The data in this study are presented in the form of tables and figures, which were analyzed using a descriptive method.

## Results

### Isolation and identification of *E. coli*

Based on the isolation and identification of 256 samples examined, the prevalence of positive *E. coli* was found to be relatively high, at 56.6% (145/256). Specifically, according to the source, the percentage of each included chicken cloaca 52.6% (92/175), fish skin 70% (42/60), pond water 100% (6/6), and farmer’s hands 33.3% (5/15).

### Double-disk ESBL confirmation test

ESBL-producing *E. coli* was identified using the double-disc diffusion test. As presented in Table-2, the results illustrated that 67.6% (98/145) isolates were ESBL-producing *E. coli*, comprising 78.3% (72/92) from chicken cloacal swabs, 47.6% (20/42) from fish skin swabs, 66.7% (4/6) from pond water, and 40% (2/5) from farmer hand swabs.

**Table-2:** Phenotype analysis results of ESBL-producing *E. coli* from 6 farms with integrated poultry-fish system.

Sample type	Number of samples	*E. coli* (%)	*E. coli* ESBL (%)
Chicken cloaca	175	92 (52.6)	72 (78.3)
Fish skin	60	42 (70)	20 (47.6)
Pond water	6	6 (100)	4 (66.7)
Farmer’s hand	15	5 (33.3)	2 (40)
Total	256	145 (56.6)	98 (67.6)

ESBL=Extended-spectrum β-lactamase, *E. coli*=*Escherichia coli*

### Molecular detection of ESBL

The *bla*TEM and *bla*CTX-M genes were identified in 100% chicken cloaca, fish skin, pond water, and farmer’s hands. *bla*SHV genes were only found in chicken cloaca (12.5%) and fish skin (40%). The *bla*OXA-48 genes were identified in chicken cloaca (13.9%), fish skin (65%), and pond water (25%). The genotype analysis of ESBL-producing *E. coli* is presented in Table-3.

**Table-3:** Genotype analysis results of ESBL-producing *E. coli* from 6 integrated poultry-fish farms.

Sample type	Number of ESBL-producing *E. coli*	Gene			

		*bla*TEM (%)	*bla*CTX-M (%)	*bla*SHV (%)	*bla*OXA-48 (%)
Chicken cloaca	72	72/72 (100)	72/72 (100)	9/72 (12.5)	10/72 (13.9)
Fish skin	20	20/20 (100)	20/20 (100)	8/20 (40)	13/20 (65)
Pond water	4	4/4 (100)	4/4 (100)	-	1/4 (25)
Farmer’s hand	2	2/2 (100)	2/2 (100)	-	-
Total	98	98/98 (100)	98/98 (100)	17/98 (17.3)	24/98 (24.5)

ESBL=Extended-spectrum β-lactamase, *E. coli*=*Escherichia coli*

## Discussion

Six farms with poultry-fish systems exhibited considerable differences in the quantities of *E. coli*. Out of 256 samples, the percentage of *E. coli*-positive samples was relatively high at 56.6% (145/256), comprising chicken cloaca 52.6% (92/175), fish skin 70% (42/60), pond water 100% (6/6), and farmer hands 33.3% (5/15). The high percentage of *E. coli* present in pond water and fish skin samples can be attributed to the contamination of chicken manure in aquatic environments, thereby facilitating infection transmission. These bacteria, which are capable of existing in water and fouling fish skin [21, 22], mirror the environmental traits of aquatic habitats [23]. According to Rocha *et al*. [24], *E. coli* in fish skin and muscle exhibited considerably distinct values, with counts of 15 (34.09%) and 4 (9.09%) concentrations, respectively.

For fish skin analysis, swab samples were collected from carp, catfish, and shark catfish. The sampled farms contained different fish species. The analysis was based on ESBL-producing *E. coli* prevalence data collected from fish in polluted aquatic environments due to antibiotic use in chickens. The pond fish of the integrated farms determined the sample type.

About 67.6% of ESBL-producing *E. coli* cells were identified through phenotype analysis. The percentages attributable to the chicken cloaca, fish skin, pond water, and farmer’s hands were 78.3% (72/92), 47.6% (20/42), 66.7% (4/6), and 40% (2/5), respectively. A Nigerian study discovered that ESBL-producing *E. coli* was prevalent among chickens, farmers, and the farm environment [25]. A lack of understanding of the cause of infection before the administration of antibiotics is the primary reason for the emergence of antibiotic-resistant bacteria. Inadequate biosecurity and poor sanitation in poultry production systems in developing countries contribute to the high dependence on antibiotics on farms for prophylactic or therapeutic purposes [26, 27].

**Figure-1:**
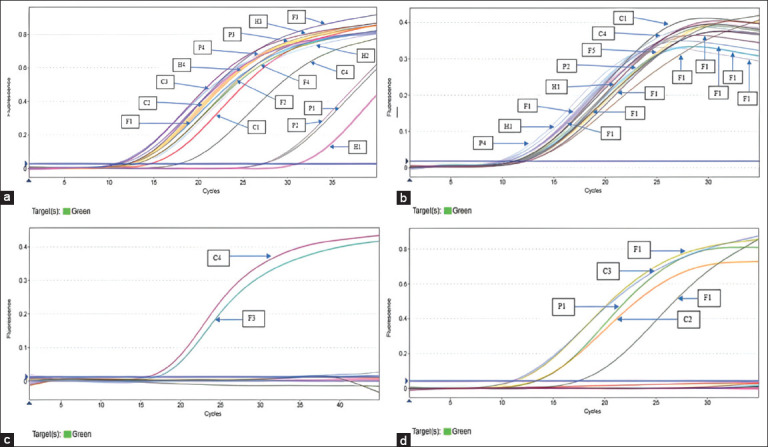
The results of testing using the quantitative polymerase chain reaction Taqman probe method showed extended-spectrum β-lactamase genes in chicken cloaca isolates, fish skin, pond water, and farmer’s hand (a) Amplification curve of *bla*TEM, (b) Amplification curve of *bla*CTX-M, (c) Amplification curve of *bla*SHV (d) and Amplification curve of *bla*OXA-48. C1-C5=Chicken cloaca isolates, FI-F5=Fish skin isolates, P1-P4=Pond water isolates, H1-H5=Farmer’s hand isolates.

The ESBL genes were detected using the qPCR Taqman Probe method for rapid and precise analysis. The method can be used in various scientific fields, specifically in screening analyses to describe the distribution of resistance genes within or between different environments [28]. The Ct values of ESBL-positive isolates were significantly below 35 after the threshold line (Figure-1). This method reliably detected ESBL genes (*bla*TEM, *bla*CTX-M, *bla*SHV, and *bla*OXA-48) in examined isolates with high sensitivity and specificity.

In chicken cloaca and fish skin samples, *bla*TEM genes were predominant. Similarly, previous studies reported that *bla*TEM was predominantly identified in many countries isolated from human, livestock, and environmental samples [29–33], commonly from ampicillin-resistant chickens and humans [34]. This study also showed that *bla*CTX-M was dominant in the chicken cloaca and fish skin isolates, in line with previous analyses by Wibisono *et al*. [35] conducted in Lidar, Indonesia, where an 80% prevalence of this disease was reported. The most common CTX-M variant found in various countries was reported to be CTX-M-15, followed by CTX-M-14, and CTX-M-27 [36]. In similar studies of poultry, such as those conducted in Bosnia and Herzegovina, *bla*CTX-M-1 and *bla*CTX-M-15 were reported to be the most dominant [37]. In Korea, CTX-M-55 and CTX-M-14 are mostly found in chicken isolates [38], whereas *bla*CTX-M-15 is dominant in 96% of broiler chicken samples in Ghana [39]. Based on an experiment conducted on fish in Saudi Arabia, *bla*CTX-M was detected in tilapia imported from Thailand and India [40].

The presence of *bla*TEM and *bla*CTX-M in farmer hand isolates was consistent with reports from studies in various countries where both genes were found in humans [41–43]. ESBL-producing *E. coli* types *bla*TEM and *bla*CTX-M were also detected in fish and pond water, in line with a study conducted in the Mekong Delta of Vietnam [44]. Previous studies by Bisi-Johnson *et al*. [45] and Chotinantakul *et al*. [46] have reported the use of *bla*TEM and *bla*CTX-M in aquatic environments. The combination of resistant bacteria spreading into water, antimicrobial accumulation, and residues in aquatic environments created selective pressures that favored the growth of antibiotic-resistant bacteria.

All isolates from the chicken cloaca, fish skin, pond water, and farmer’s hand were positive for *bla*TEM and *bla*CTX-M. ESBL bacteria in manure-contaminated pond water beneath chicken coops contribute to the spread of ESBL-producing *E. coli* in fish. Animal contact with humans and the environment facilitates the dissemination of the × into multiple populations [47]. Our results do not provide a deeper understanding of how *E. coli* ESBL in humans originates from chickens and the farm environment. In-depth genetic studies, using whole-genome sequencing are essential for a comprehensive understanding of the relationships between genes in animals, the environment, and humans.

In the Philippines [48], Brazil [49], and Egypt [50], studies have reported the occurrence of *bla*SHV in isolates of chicken origin. In Lidar, Indonesia, no chicken samples tested positive for the *bla*SHV gene [51]. This difference occurred because of several factors affecting the spread of bacteria, such as geographical location, population density, hygiene, and antibiotics [52]. In Malaysia, *bla*OXA-48 was identified in chicken isolates [53], whereas in India, both *bla*SHV and *bla*OXA genes were detected in fish isolates [54].

Abrar *et al*. [55] have reported the absence of *bla*SHV and *bla*OXA-48 in human isolates from Pakistan. In contrast, a study from Mecklenburg-Western Pomerania, Germany, identified *bla*CTX-M, *bla*TEM, and *bla*OXA in human isolates but did not report the presence of *bla*SHV [56].

The *bla*SHV and *bla*OXA-48 genes were identified in chicken and fish isolates based on the results. *bla*SHV was absent in pond water, whereas *bla*OXA-48 was detected in a single sample. This variation can be attributed to observations in the field, where water conditions were constantly flowing and the size of the pond was larger than that of the coop. Therefore, it is possible that the *bla*SHV and *bla*OXA-48 genes were not identified in the pond water samples examined. The presence and spread of the *bla*SHV and *bla*OXA-48 genes require further exploration because there is a possibility of vertical or horizontal transfer between bacteria spreading in the environment. The identification of both genes in fish could be attributed to the extended period in which the fish were kept in the pond, ranging from 4 to 6 months before being harvested for sale, leading to prolonged exposure to water containing ESBL-producing *E. coli*.

The identification of ESBL-producing *E. coli* genes in chicken cloaca, pond water, fish, and farmer’s hand showed the possibility of chicken as a reservoir of antibiotic-resistant bacteria. This phenomenon poses a significant risk to human health through direct contact with the environment and the food chain. ESBL-producing *E. coli* genes found in fish isolates may transfer resistance to other aquatic species. Genes encoded in plasmids are easily transmitted between species horizontally through mobile genetic elements and vertically by self-division [57]. The high incidence of ESBL-producing *E. coli* in healthy animals requires immediate control because the strains and resistance genes can spread widely to humans through the environment and food chain.

## Conclusion

The *bla*TEM and *bla*CTX-M genes (100%) were the most dominant genes found in the cloaca of chickens, fish, pond water and farmers’ hands, followed by *bla*OXA-48 and *bla*SHV at 24.5% and 17.3% respectively over the period one year’s time. interconnected poultry trial fish farming operations. ESBL-producing *E. coli* in chickens can transfer resistance genes to the aquatic environment. This transition could endanger other aquatic species and food chains, as well as potentially threaten human health. To develop more effective surveillance and control strategies in the future, further studies of resistance patterns and genetic studies are needed for a better understanding of the genetic relationships between genes found in several populations in the same environment.

## Authors’ Contributions

KSH, AS, DWL, and HP: Coordinated the study, developed ideas, and designed the study. KSH: Performed sample collection and testing, data analysis, and manuscript writing. PR: Sample testing and data analysis. All authors have read, reviewed, and approved the final manuscript.
